# Energizing Community Health Improvement: The Promise of Microgrants

**Published:** 2005-10-15

**Authors:** Mary Bobbitt-Cooke

**Affiliations:** Office of Healthy Carolinians/Health Education, Division of Public Health, North Carolina Department of Health and Human Services

## Abstract

**Background:**

The Healthy Carolinians community microgrants project provided microgrants to community-based organizations (CBOs) across North Carolina. These grants were made to serve as a catalyst to engage the CBOs in health promotion activities that addressed *Healthy People 2010* objectives. The purpose of this initiative was to increase the awareness of *Healthy People 2010* objectives, mobilize resources, and create new partners in community health improvement.

**Context:**

In 1993, Healthy Carolinians, a statewide network of public–private partnerships, was established at the county level to address North Carolina's health objectives that aligned with national *Healthy People 2010* objectives. This network of Healthy Carolinians partnerships provided the vehicle for distributing the microgrants.

**Methods:**

Funding was distributed to 32 Healthy Carolinians partnerships that, in turn, awarded 199 microgrants ($2010 each) to CBOs to address state and national health objectives. Each CBO selected its own objectives based on *Healthy People 2010* objectives and designed its own interventions. Surveys of the CBO project managers and final reports were used for evaluation. A survey of the Healthy Carolinians partnership coordinators provided additional insight.

**Consequences:**

Of the 199 surveys mailed to CBOs, 153 (77%) responded. Nearly half (43.7%) of the microgrants were used to focus on three major health risk factors: 27.1% on physical activity and fitness, 13.1% on nutrition and overweight, and 3.5% on tobacco use. At the end of the project, 96.1% of the respondents reported that they were familiar with the *Healthy People 2010* objectives. Final reports showed that an estimated 52,739 hours of CBO staff and volunteer time were contributed to microgrant programs. All Healthy Carolinians partnership coordinators responded to a survey; 100% stated that they had new access to priority populations within their community.

**Interpretation:**

The Healthy Carolinians microgrant project demonstrated a cost-effective, alternative approach to funding community-based health promotion and injury control activities. This model was decentralized, so it empowered communities and CBOs to be responsible for community health improvement. Public health professionals with limited funds should consider this alternative approach, which mobilized existing community organizations and effectively addressed national and state health objectives.

## Background

In 2001, the Office of Healthy Carolinians/Health Education (OHC/HE) within the Division of Public Health, North Carolina Department of Health and Human Services, was awarded a $510,000 grant from the Office of Disease Prevention and Health Promotion (ODPHP), U.S. Department of Health and Human Services. The purpose of the Healthy Carolinians microgrant project was to increase public awareness of *Healthy People 2010* objectives and broaden participation of community-based organizations (CBOs) in innovative efforts to promote healthy behaviors, access to care, and other projects to support national health objectives (1-3).

This initiative was designed to use existing community organizations rather than to build infrastructure at local and state health departments. Instead of sending funding to a community public health agency to implement a community health promotion project, this initiative sought to determine whether small grants ($2010) to CBOs could serve as a catalyst for increasing awareness and mobilizing resources to address national *Healthy People 2010* objectives. By investing small grants in CBOs, critical capital was provided in areas where people learn, work, play, worship, and live. These funds, applied to health improvement projects, could potentially empower small agencies and groups to respond to the health needs of their community.

Chronic diseases such as heart disease, stroke, cancer, and diabetes are among the most prevalent, costly, and preventable of all health problems (4). Unintentional injuries, which are preventable, represent the leading cause of death for people aged 1 year to 34 years; about 50% of unintentional injuries are related to motor vehicles (5). The challenge was to determine whether microgrants could effectively stimulate communities to promote health and safety, thereby preventing chronic disease and injury. The idea of providing microgrants to CBOs for community health promotion is consistent with the 1997 report from the Institute of Medicine, which called for a community health improvement process mobilized by a coalition that is centered in the community and includes groups interested in health outcomes (6).

## Context

Since 1991, North Carolina has addressed the national *Healthy People* objectives through the Healthy Carolinians initiative, a network of community-based, public–private partnerships across North Carolina. By executive order in 1991, the Governor's Task Force for Healthy Carolinians was established to develop North Carolina's health objectives and ensure that these objectives aligned with national *Healthy People* objectives (7). In 1992, the health objectives for North Carolina were published, and the Governor's Task Force challenged all counties in North Carolina to mobilize community resources to address the problems identified in the state and national objectives. The Governor's Task Force believed that if communities determined their own health priorities, they would mobilize to address them (8,9). The Healthy Carolinians network of partnerships placed resources, decision making, and accountability where health decisions are created and supported — in the community.

Across North Carolina, communities have embraced the idea that they can decide what their health challenges are, how to fix their problems, generate or redirect resources, and implement their own community-devised solutions. Healthy Carolinians partnerships, now in more than 90 counties, represent health departments, hospitals, schools, churches, businesses, media, human service organizations, and civic groups. The Governor's Task Force certifies each partnership using a rigorous set of criteria and holds the partnerships to standards that are proven to support successful health outcomes (10). Within this context, the concept of microgrants was tested in North Carolina through the Healthy Carolinians microgrant project.

## Methods

The Healthy Carolinians microgrant project included the following three overarching goals:

1. To provide microgrants ($2010 each) to a wide variety of CBOs conducting activities related to *Healthy People 2010* objectives
2. To demonstrate the advantages of the statewide Healthy Carolinians network
3. To evaluate the concept of using microgrants as an alternative method for increasing awareness and mobilizing resources for addressing *Healthy People 2010* objectives

In 2002, the OHC/HE distributed $439,790 to 32 Healthy Carolinians partnerships representing 35 counties in North Carolina (an average of $13,743 per Healthy Carolinians partnership). Each partnership receiving the funding participated in an orientation video conference and received a toolkit that included the following guidelines for administering the project: a description of methods for soliciting applications for the microgrants (e.g., sample press releases in English and Spanish, advice on how to run a community meeting); microgrant applications and instructions for completing the applications in English and Spanish; guidelines and criteria for selecting CBOs from the applicant pool; reporting forms; and background information on *Healthy People 2010*. Each of the 32 Healthy Carolinians partnerships used the same application form and selection criteria. Partnerships were evaluated on uniformity in selecting grantees and reporting information. The partnerships were given $200 for each microgrant that they administered to cover expenses for meetings, postage, site visits, and other items. Four to nine microgrants were awarded in each of the participating counties for a total of 199 microgrants.

Healthy Carolinians partnership coordinators informed local CBOs about the availability of microgrants through electronic mailing lists, print and broadcast media (including paid advertising and public service announcements), and word-of-mouth. Healthy Carolinians partnership coordinators held community meetings to explain the microgrant project and help CBOs understand the application process. A panel established by each partnership made award decisions.

More than 275 CBOs applied for a microgrant. Microgrants were awarded to CBOs representing organizations that traditionally work with local public health departments (e.g., churches, schools, cooperative extensions, preschool centers) and organizations that are not traditional partners with the local public health department (e.g., Boy Scouts, neighborhood and community development organizations). Large CBOs (e.g., United Way agencies) did not apply for these small grants. The pool of proposals came primarily from small organizations, with more than one third (39%) indicating that this was the first time they had ever applied for a grant.

Because the CBO applicants decided the health objectives on which to focus, they determined the health priorities of the Healthy Carolinians microgrant project. The project allowed CBOs to set their own project goals using *Healthy People 2010* objectives for guidance. Table 1 shows that nearly half (43.7%) of the microgrants were used to focus on the three major health risk behaviors outlined by *Healthy People 2010*: 27.1% of the microgrant recipients addressed physical activity, 13.1% addressed nutrition and overweight, and 3.5% addressed tobacco use among children and youth. Table 1 reports the types of objectives selected by the CBOs. The populations addressed most often by the microgrant programs were children (52%), racial or ethnic minorities (36%), and low-income individuals (33%). (Categories may overlap.)

Each microgrant program was designed to run for 9 months; none was expected to provide health outcome data to the OHC/HE. Thus, the evaluation of the Healthy Carolinians microgrant project was not designed to measure health outcomes. Instead, the evaluation of the microgrant project focused on 1) testing the effectiveness of the Healthy Carolinians partnerships in distributing microgrants to a wide variety of CBOs, 2) identifying components of the model that could be used by other states, and 3) analyzing the benefits of using existing community infrastructure (CBOs) for community health improvement. Evaluation data were attained through surveys and final reports. CBO project managers and Healthy Carolinians coordinators were asked questions about their experience with the microgrant project. The feedback was used to answer the initial question of the project: can microgrants serve as a catalyst for increasing awareness and mobilizing resources for addressing *Healthy People 2010* objectives?

### Survey of CBOs

A two-page survey with a self-addressed stamped envelope was mailed to the program directors of all 199 microgrant-recipient CBOs after the programs were completed. Eleven survey questions addressed familiarity with the objectives of Healthy Carolinians and *Healthy People 2010* and asked respondents to rate their satisfaction with such aspects of the microgrant project as the application process, reporting requirements, ease of recruiting volunteers, and working relationship with Healthy Carolinians partnerships. Seven other yes-or-no questions asked about future relationships with Healthy Carolinians partnerships, current and future experience with grant writing, mobilizing volunteers, and continuing activities beyond the $2010 microgrant project.

### CBO final reports

Each CBO was required to submit a uniform final report. This report requested information about expenditures, number of staff engaged in the microgrant activities, number of volunteers involved in the project, and number of hours that both staff and volunteers spent on the project. Through open-ended questions, the final report also provided an opportunity for CBOs to comment on their experience, describe achievements, and discuss their plans (if any) to continue the project and their relationship with the Healthy Carolinians partnerships after the project expired.

### Survey of Healthy Carolinians partnership coordinators

The 32 Healthy Carolinians coordinators who managed the community microgrants project also completed a survey at the end of the project. They could complete the survey online or print the survey, complete a hard copy, and fax or mail it to the OHC/HE. Survey questions were ranked on a 4-point scale with 1 indicating strongly agree; 2, agree; 3, disagree; and 4, strongly disagree. The survey asked questions about the following:

- Orientation and support provided by the OHC/HE to the Healthy Carolinians coordinator during the microgrant project
- Management of the microgrant project (e.g., adequate time to promote the microgrants in the community)
- Rules about CBO eligibility being clear and fair
- Guidance provided in the microgrant toolkit
- Adequacy of the $200 allowed for administration of the microgrant
- Help they provided to the CBOs
- How their Healthy Carolinians partnership benefited from the microgrant project (Did the microgrant project diversify the partnership's membership? Did it improve access to priority populations? Did it provide the partnership positive exposure in the community?)

The survey of Healthy Carolinians coordinators also provided an opportunity for coordinators to comment on the project as a whole or any specific part as well as to make recommendations for changes.

## Consequences

### CBO survey results

Of the 199 questionnaires mailed, 153 (77%) were returned. Overall, the CBO respondents provided positive feedback about the microgrant project (Table 2).

### CBO final report results

The final reports from all 199 CBO microgrant projects provided insights into the ingenuity and determination of the CBOs during the project. The survey asked CBO microgrant recipients to describe their achievements. The following is a sample of the achievements, with the corresponding *Healthy People 2010* objectives provided in parentheses (3). This list demonstrates that a significant amount of work was accomplished with relatively small investments used strategically in communities.

- The Crossroad Sexual Assault Response and Resource Center reached 3818 people through its program and has 20 new volunteers working with it (Objective 15-36).
- Carson Community Development Center leveraged the $2010 to raise more than $90,000 in community donations and grants to build a walking track and multisport playing field at a local elementary school. This walking track is close to an industrial park and a residential home for older adults; it is used by a wide range of children and adults (Objectives 22-4, 22-6, and 22-12).
- Phi Beta Kappa used a puppet skit to educate 2200 youth about the health risks of tobacco and will continue this program (Objective 27-2).
- South Macon Elementary School Parent–Teacher Organization provided fluoride sealants to 1887 children (Objective 21-8).
- Pitt Council on Aging delivered 673 meals to 26 shut-in seniors for 6 months and identified new funds to continue this initiative (Objective 19-18).
- Hispanic Coalition of Salisbury, with assistance from the sheriff's department, provided and installed car seats for more than 50 Hispanic families and obtained a $1000 grant for additional car seats from a local store (WalMart), which indicated that it will continue to support this program (Objectives 15-20, 15-15, and 15-17).
- The Ashe County 4-H Club established a physical activity program for home-schooled children — the first such program in the state (Objectives 22-6 and 22-12).
- The Lake Users Association, Swain County, purchased a boat and other equipment to clean a lake that was polluted after a major flood. It is now clean enough for recreational purposes (Objective 8-8).

Several CBOs bought physical activity equipment for their churches, youth agencies, or own organizations; provided a health fair for their community; or offered training sessions.

The final report provided the opportunity for the CBOs to identify challenges as well. The themes identified as challenges or barriers to the project were weather, scheduling of project activities, staffing, time constraints, funding delays, excessive reporting requirements, recruiting volunteers, and finding Spanish-language materials. The weather was a particular challenge for projects in the mountains. The unusually wet winter and spring in North Carolina (2002–2003) led to many outdoor project delays. The 9 months allotted for project implementation was not enough for some projects. In addition, the North Carolina Department of Health and Human Services had problems distributing funds at the beginning of the project. The problems were eventually resolved, but funding was delayed initially. Finally, because funds were distributed before expenditures were made, the North Carolina Department of Health and Human Services required the CBOs to file monthly expense statements.

There were only a few responses related to lessons learned, such as, "I would make a weather contingency plan" and "I should have used the funds to leverage other funds. Now I have to start from scratch to find new funds to keep the project going." Most responses to the question on what CBOs would do differently were related to project administration: for example, allowing more time for planning, increasing staff and volunteers, offering their program to a wider audience, ordering materials earlier, advertising and promoting the program, and involving participants in selecting equipment.

The CBOs were asked how the Healthy Carolinians partnerships helped them in their work. This question was important to determine whether the microgrant project could be replicated and, if so, what type of support would be needed. The following are the most often reported services provided by the Healthy Carolinians partnerships:

- Networking with other nonprofit agencies
- Providing skills on how to leverage microgrants to secure other funds
- Providing resource materials and information (e.g., Internet sites, funding sources)
- Planning and evaluating projects
- Promoting the project and CBO, using newspapers and radio to increase visibility
- Increasing awareness about community health issues
- Supporting administrative functions (e.g., completing reporting forms, purchasing supplies, identifying vendors)

The final reports also provided information about the amount of resources that each CBO contributed to its own project. The CBOs were asked to indicate how many of their staff members had participated in the project, how many volunteers they had recruited, and the approximate number of hours that both staff members and volunteers contributed. The following was reported:

- 2486 CBO staff members worked on microgrant programs.
- 4409 volunteers were recruited.
- 52,739 hours (estimated) of CBO staff and volunteer time were spent on microgrant programs.

When the 52,739 hours are multiplied by $16.54/hour — the average value of a volunteer hour according to the nonprofit research group Independent Sector (11) — the total CBO in-kind labor contribution to the microgrant project is valued at $872,303, almost a 200% return on the $439,790 awarded in microgrants.

### Healthy Carolinians partnership coordinator survey results

Twenty-eight of 32 (88%) Healthy Carolinians coordinators responded to the survey. Twenty-one of 28 (75%) coordinators indicated that they would want more time than the 2 months allotted to adequately advertise the funding opportunity and recruit CBOs to complete an application. When asked about how the microgrant initiative affected their Healthy Carolinians partnerships, the coordinators responded as follows:

- 93% (26 of 28) stated that the microgrant project helped to diversify the partnership's membership.
- 100% (28 of 28) stated that they had expanded their access to priority populations within their community.
- 96% (27 of 28) stated that the microgrant project helped their partnership gain positive community exposure.
- 96% (27 of 28) stated that, given the opportunity, they would like to participate in another microgrant project.
- 89% (25 of 28) thought that the microgrant toolkit was useful.
- 43% (12 of 28) stated that the proposal reviewers needed guidance, training, or both to review the CBO proposals.
- 14% (4 of 28) stated that rules about allowable expenses were not clear.

When asked how they helped the CBOs during the funding period, the most common responses from the Healthy Carolinians coordinators were as follows:

- Linking the CBO with other agencies and resources in the community
- Identifying funding sources to help the CBO continue their projects
- Providing publicity and media exposure
- Helping with project planning and budgeting

## Interpretation

The Healthy Carolinians microgrant project was successful in distributing small grants of $2010 to 199 CBOs. The CBOs self-selected their initiatives and designed their own projects. Almost half (43%) addressed the three major risk behaviors that contribute to chronic disease — physical inactivity, poor nutrition and overweight, and tobacco use. This finding demonstrates that many partners already in place at the community level are aware of major health risk behaviors and are eager to mobilize themselves and their communities to address these problems. The answer to the question, "Is this an effective, alternative method to funding community health promotion?" is clearly yes. With an average of $13,743 for each partnership ($439,790 divided by the 32 Healthy Carolinians partnerships) with an average of six unique projects, this approach to community health improvement appears to be highly cost-effective. This is an alternate funding mechanism to states and local public health agencies to spread limited funds across the community.

The Healthy Carolinians microgrant project was a catalyst for increasing awareness and mobilizing underused and previously untapped community resources. These small investments stimulated health promotion and injury control activities linked to *Healthy People 2010* objectives. The small CBOs were creative with their funds, and many were able to reach pockets of the community that are priority populations. The skill with which the CBOs mobilized additional resources — both funds and volunteer help — yielded a high return on the original modest investment.

The Healthy Carolinians partnerships were perfectly positioned to be grant makers in their communities by providing the structure and support for microgrants. In most cases, Healthy Carolinians partnerships and CBOs continued their relationships after funded programs were completed. The relationship between the CBOs and the Healthy Carolinians partnerships was mutually beneficial. Not only did CBOs have access to new funds but the project managers also learned new skills (e.g., proposal development) and networked with other CBOs in their community.

The Healthy Carolinians microgrant project demonstrated a cost-effective, alternative approach to funding health promotion and injury control activities at the community level. This model was decentralized, and it empowered communities and CBOs to be responsible for community health improvement. Public health professionals with limited funds should seriously consider this alternative approach, which mobilizes existing community organizations to address national and state health objectives.

## Figures and Tables

**Table 1:** Microgrant Awards by Focus Area Established by *Healthy People 2010*, Healthy Carolinians Microgrant Project, North Carolina, 2002–2003

**% Microgrants (No.)**	**Focus Areas**
27.1 (54)	Physical activity and fitness
13.1 (26)	Nutrition and overweight
13.1 (26)	Injury and violence prevention
7.0 (14)	Sexually transmitted diseases
5.0 (10)	Access to health care
4.0 (8)	Substance abuse
3.5 (7)	Tobacco use
3.5 (7)	Health communication
23.6 (47)	Other^a^

a Cancer, 1.5% (3 grants); diabetes, 3.0% (6 grants); disability, 2.5% (5 grants); educational and community-based programs, 1.5% (3 grants); environmental health, 1.5% (3 grants); family planning, 2.5% (5 grants); heart disease, 2.5% (5 grants); immunization, 0.5% (1 grant); maternal and infant health, 1.0% (2 grants); oral health, 2.4% (5 grants); respiratory diseases, 2.0% (4 grants).

**Table:** 

**Strongly Agree or Agree(%)**	**Disagree or Strongly Disagree(%)**	**Statement**
98.7	1.4	Overall, my organization had a satisfactory experience with the microgrant project.
97.4	2.6	My microgrant project achieved its goals.
96.1	3.9	My organization is familiar with Healthy People/Healthy Carolinians 2010 objectives.
97.3	2.7	Working with our Healthy Carolinians partnership has furthered my organization's interest in community health improvement.
98.0	2.0	My Healthy Carolinians partnership was helpful with the application process.
93.9	6.2	Directions for application and completing the budget for the microgrant were easy to understand.
87.7	0	Our evaluation of the project gave us useful information.^b^
76.8	23.2	It was fairly easy to enlist volunteers to help with our microgrant project.
NA^c^	NA^c^	Enough time was allotted to complete the application.
NA^c^	NA^c^	The number of required reports was excessive.
NA^c^	NA^c^	The delayed funding forced us to change the original plan.

**Table:** 

**Yes (%)**	**No (%)**	
89.5	2.6	Will your organization continue to work with your Healthy Carolinians partnership?
93.5	5.2	Will the grant activities continue beyond the $2010 funding?
84.9	11.8	Did the microgrant help your organization gain visibility in the community?
25.0	73.6	Was the $2010 grant your first experience in receiving a grant?^d^
94.2	0.0	Will your organization apply for another grant to continue the work of your project?^e^
55.8	42.3	Did the grant increase volunteer service opportunities for your organization?^d^
92.3	5.8	Did your organization improve its capacity to serve priority populations?^d^

a 153 of 199 (76.9%) returned the questionnaires.

b 134 of 153 respondents answered this question.

c NA indicates not applicable. Fewer than 5% of respondents answered this question; results are not reported.

d 150 of 153 respondents answered this question.

e 144 of 153 respondents answered this question.
